# Functional visual acuity after implantation of diffractive extended depth-of-focus intraocular lenses using an echelett optics

**DOI:** 10.1186/s12886-021-02189-7

**Published:** 2021-12-05

**Authors:** Toshihiro Sakisaka, Keiichiro Minami, Keita Takada, Yosai Mori, Kazunori Miyata

**Affiliations:** Miyata Eye Hospital, Miyakonojyo, Miyazaki Japan

**Keywords:** Extended depth-of-focus intraocular lens: functional visual acuity, Visual function, Contrast sensitivity

## Abstract

**Background:**

The prospective comparative case series aimed to evaluate all-distance visual acuity, contrast sensitivity, and functional visual acuity (FVA) of eyes with diffractive extended depth-of-focus (EDOF) intraocular lenses (IOLs) using an echelett optics and monofocal IOLs with the same platform.

**Methods:**

Diffractive EDOF and monofocal IOLs were implanted in 27 eyes of 27 patients each. At 3 months after implantation, all-distance visual acuities at distances of 0.3, 0.5, 0.7, 1, 2, 3, and 5 m were measured under distance-corrected. Static visual function was also examined using photopic contrast sensitivity and area under the logarithmic contrast sensitivity function (AULCSF). Dynamic visual function was examined with FVA, and mean FVA value, visual maintenance ratio (VMR), mean response time, and number of blinks were evaluated. These outcomes were compared between the two IOLs.

**Results:**

The mean distance-corrected visual acuities were better at distances of 0.7 m or nearer in eyes with EDOF IOLs. There was no difference in the contrast sensitivities (*P* > 0.22). In the FVA results, no difference was found in mean FVA and VMR (*P* > 0.68).

**Conclusion:**

The static and dynamic evaluations of postoperative visual functions demonstrated that the visual function of eyes with EDOF IOLs under photopic and distance-corrected conditions was comparable to eyes with monofocal IOLs.

**Supplementary Information:**

The online version contains supplementary material available at 10.1186/s12886-021-02189-7.

## Background

The diffractive extended depth-of-focus (EDOF) intraocular lens (IOL) Symfony® (Johnson & Johnson Surgical Vision, Santa Ana, CA) is designed for presbyopia correction by utilizing an echelle optics, and enhances postoperative visual acuity between far and intermediate distances with the least photic phenomena [1]. In eyes with binocular implantations, 20/20 or better visual acuities were obtained at distances of 0.7 m or longer [2], and the range could be expanded with the micro monovision technique [1–3]. Owing to compensation of chromatic aberrations [4], the postoperative contrast sensitivities are improved to be comparable with the use of monofocal IOLs [5, 6]. The visual function has been assessed by using a visual acuity chart with high-contrast optotypes and a contrast sensitivity chart. Such conventional examinations are static evaluations only, so that it is important to evaluate dynamic property for assessing the quality of vision. Furthermore, contrast sensitivity test measures in a step to 0.12 to 0.15 logarithm unit [7], so that it is hard to identify slight differences .

Functional visual acuity (FVA) testing was developed to evaluate dynamic changes in visual functioning [8–11]. FVA has been also used for detecting slight impairment of visual function due to posterior capsule opacification (PCO) [12] and hydration-related subnanometer vacuoles in the IOL surface layer [13], which could not be detected with conventional contrast sensitivity testing. It was anticipated that FVA examination would be effective to precisely evaluate visual function after EDOF IOL implantation. To our knowledge, FVA of eyes with EDOF IOLs has not been assessed. This comparative prospective study aimed to compare postoperative distance visual function, including FVA, in the use of EDOFs and monofocal IOLs.

## Methods

### Participants

The prospective comparative study was approved by the ethics committee of Miyata Eye Hospital (identifier: CS-295) and adhered to the tenets of the Declaration of Helsinki. Written informed consent was obtained from all subjects before enrollment. Patients who were aged between 60 and 69 years and would have no postoperative complications and residual astigmatism of 1.25 Diopter (D) or less, were recruited. Exclusion criteria were previous ocular surgery and disease influencing visual function except for cataract, such as chronic or recurrent uveitis, acute ocular disease or external/internal infection, diabetes with retinal changes, glaucoma, exfoliation syndrome, pathological miosis, keratoconus, corneal endothelial dystrophy, and abnormality in the capsule, zonule, or pupil. Eyes with postoperative corrected distance visual acuities (CDVAs) below 20/20 were also excluded.

The subjects were divided into two groups according to the implanted IOL, EDOF ZXR00V or monofocal ZCB00V (Johnson & Johnson Surgical Vision). Patients selected the IOL according to their preferences on postoperative vision. For patients preferring vision between far and intermediate distances with less use of spectacles, EDOF IOL was recommended. For other patients who were not interested in presbyopia correction or were uncomfortable with the photic symptoms associated with the use of EDOF IOL, monofocal IOL was recommended. Due to differences in the postoperative outcomes and surgery cost, randomization was not conducted. With sufficient explanations of the benefits and risks of both types of IOLs, the implanted IOLs were determined.

The sample size for each group was determined to be 17 patients or more. This sample size was required to detect differences in the FVA values of 0.15 logMAR (between the next and 2nd steps) with a significance level of 0.05 and a detection power of 0.90 when the SD of FVA was 0.13 logMAR [13].

### Intraocular lenses

One-piece, violet-light blocking, hydrophobic acrylic, diffractive EDOF IOLs Symfony® ZXR00V were implanted. The IOL optics of 6.0-mm diameter had aspheric design on the front surface, continuous sharp optic edges on the posterior, and anteriorly shifted haptics. The EDOF function was produced with an echelett optics; the 1st-order diffraction formed the distance focus and the 2nd-order diffraction added + 1.75 D power to extend the focus range. Consequently, preferred visual acuity is provided from far to 0.7 m [4]. With biometry data obtained with a swept source biometer OA-2000 (Tomey Corporation, Nagoya, Japan), the powers of the IOLs were determined for postoperative emmetropia. As a control, monofocal IOLs ZCB00V. The both IOLs had the identical material and platforms except for the echelle optics. The postoperative refractions were intended for between 0.0 D and − 0.5 D. After removing cataracts using a phacoemulsification and aspiration technique, both IOLs were implanted completely within the capsules using the inserter system.

### Postoperative examinations

At 3 months after surgery, CDVA, manifest refraction spherical equivalent (MRSE), and contrast sensitivity were measured. For eyes with EDOF IOLs, the CDVA was measured in usual manner, then the spherical powers increased until the corrected visual acuity decreased from the best-corrected values; the power before the decrease was recorded as the MRSE [2]. For all eyes, distance-corrected visual acuities at distances of 0.3, 0.5, 0.7, 1, 2, 3, and 5 m were also examined using the all-distance vision tester AS-15 (Kowa, Nagoya, Japan) [14]. At each distance, a Landolt ring was randomly displayed, and the best visual acuity was measured. All visual acuity data were converted to the logarithm of the minimum angle of resolution (logMAR) for analysis.

After best correcting distance visual acuity, photopic contrast sensitivity at 1.5, 3, 6, 12, and 18 cycles per degree (cpd) was measured using an Optec6500 (Stereo Optical, Chicago, IL) under photopic illumination (85 cd/m^2^). From the measured data, the area under the logarithmic contrast sensitivity function (AULCSF) [15] was also calculated.

Postoperative FVA was measured monocularly using an AS-28 (Kowa), as described previously [8–10, 12, 13]. Under distance-corrected conditions, static visual acuity was initially measured using the Landolt ring chart, which was automatically shown in the screen in the equipment. Subjects delineated the orientation of the ring by handling the joystick. Optotype size was changed in single steps depending on the subject’s responses: the optotype was enlarged when the patient’s response was incorrect or reduced for the correct response. When there was no response within 2 s, an error was recorded, and the optotype was enlarged. After testing for 60 s., the FVA value, visual maintenance ratio (VMR), mean response time, and number of blinks were provided. The FVA value was the mean of the measured visual acuities, and VMR was defined as the FVA value divided by the start visual acuity. The response time, which was the time from a change in optotype size until the correct response, was averaged. As FVA testing results were affected with dry eye syndrome (DES) [8, 9, 16], incidences of superficial punctate keratitis (SPK) and the use of topical DES treatments were recorded.

### Statistical analysis

For subjects bilaterally implanted, eyes with better CDVA were selected for analysis; when both CDVAs were equal, the right eye was chosen. Differences in CDVA, distance-corrected visual acuities in the range from 0.3 m to 5 m, and FVA values were examined using the Mann-Whitney test, since their distributions were inherently non-Gaussian. For VMR. mean response time, numbers of blinks, contrast sensitivity, the normality was examined using the Shapiro-Wilk test: t-test was used when the normality was confirmed, otherwise the Mann-Whitney test was used. The influence of DES were examined by comparing the FVA results between eyes with and without DES treatments. *P* > 0.05 was considered a significant difference.

## Results

There were 27 eyes from 27 patients receiving the EDOF and monofocal IOLs, each. The demographic data of the subjects are shown in Table 1. Although the postoperative MRSE was myopically shifted in eyes with EDOF IOLs (*P* = 0.0054, t-test), the mean difference of 0.27 D was close to minimum step of added refractions (0.25 D), which was considered as clinically negligible. There was no difference in age, axial length, mean keratometry, or CDVA.

**Table 1:** Demographic data of the subjects for analysis

IOL	EDOF	Monofocal	*P* value
	27 eyes	27 eyes	
Age, year	69.0 (4.2) [62 – 79]	70.9 (4.5) [62 – 78]	0.10*
Axial length, mm	23.8 (1.5) [21.9 – 28.2]	23.9 (0.9) [22.7 – 26.1]	0.68*
Mean keratometry, D	44.5 (1.5) [41.0 – 47.8]	43.8 (1.4) [40.9 – 46.3]	0.08*
CDVA, logMAR	−0.16 (0.05) [− 0.18 – 0.00]	−0.16 (0.04) [− 0.18 – − 0.08]	0.95^#^
MRSE, D	−0.37 (0.37) [− 1.25 – 0.25]	−0.10 (0.36) [− 1.00 – 0.63]	0.010*

Mean (standard deviation) [range]

*: unpaired t-test, #: Mann-Whitney test, *IOL* intraocular lens, *D* diopter, *CDVA* corrected distance visual acuity, *MRSE* manifest refraction spherical equivalent, *EDOF* extended depth of focus

**Fig. 1:**
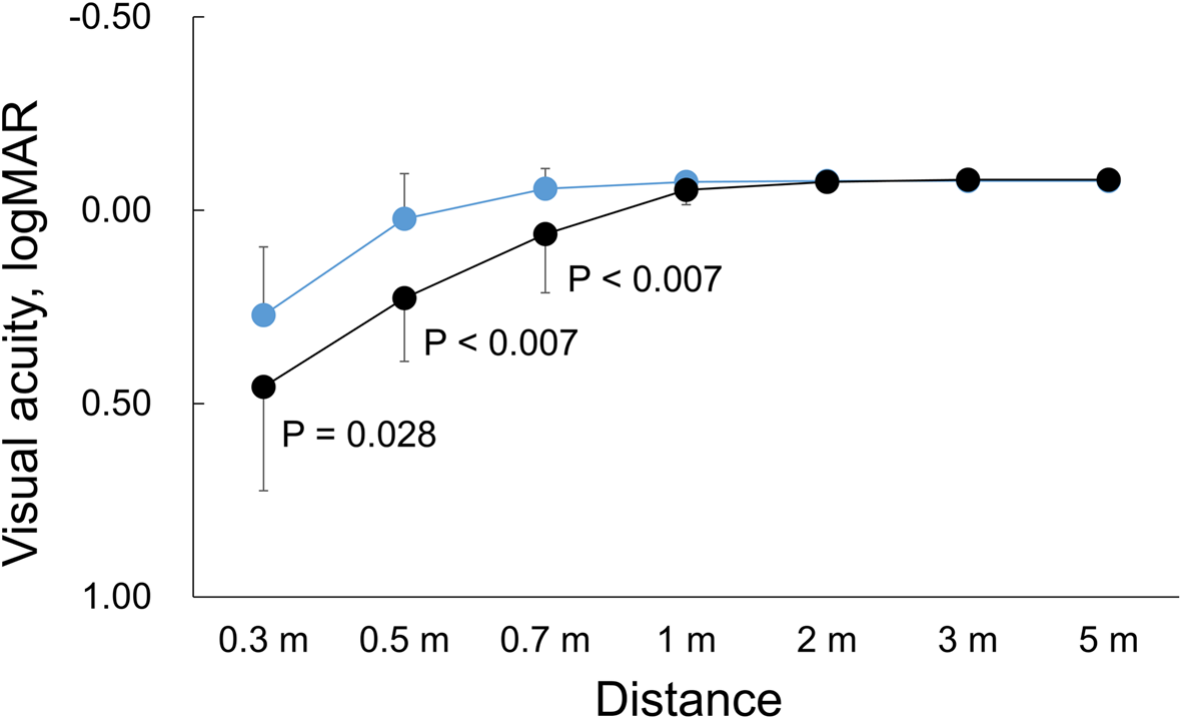
Distance-corrected visual acuities at distances of 0.3, 0.5, 0.7, 1, 2, 3, and 5 m of eyes with EDOF (blue) and monofocal (black) IOLs. *P* values denote significant differences between 2 IOLs (unpaired t-test with Holm correction)

Figure 1 shows distance-corrected visual acuities at distances of 0.3, 0.5, 0.7, 1, 2, 3, and 5 m. The mean visual acuities in eyes with EDOF IOLs were 20/20 or better at distances of 0.7 m and farther and decreased at 0.5 and 0.3 m (mean in Snellen: 20/21 and 20/37, respectively). In the use of monofocal IOLs, visual acuities of 20/20 or better were obtained beyond 1 m. Between the 2 IOLs, there were significant differences at distances of 0.7 m or nearer (*P* < 0.028, Mann-Whitney test with the Holm correction).

**Fig. 2:**
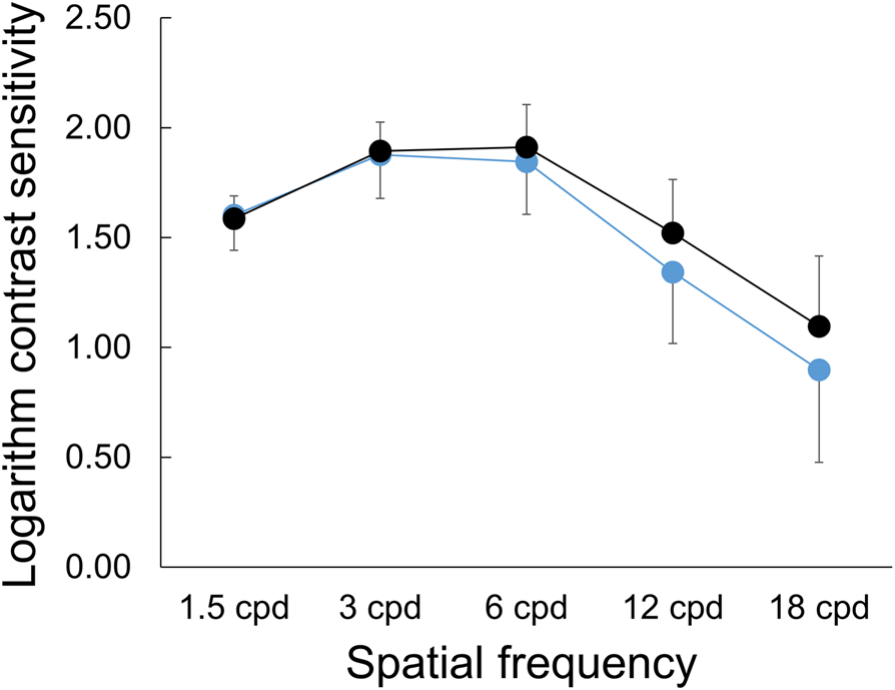
Photopic contrast sensitivities of eyes with EDOF (blue) and monofocal (black) IOLs

Figure 2 shows photopic contrast sensitivities of eyes with EDOF and monofocal IOLs. There was no difference at any spatial frequency (*P* > 0.22, Man-Whitney test with the Holm correction). The mean AULCSF in eyes with EDOF and monofocal IOLs was 1.78 and 1.86 (SD: 0.23 and 0.16), respectively, with no significant difference (*P* = 0.31, Man-Whitney test).

**Fig. 3:**
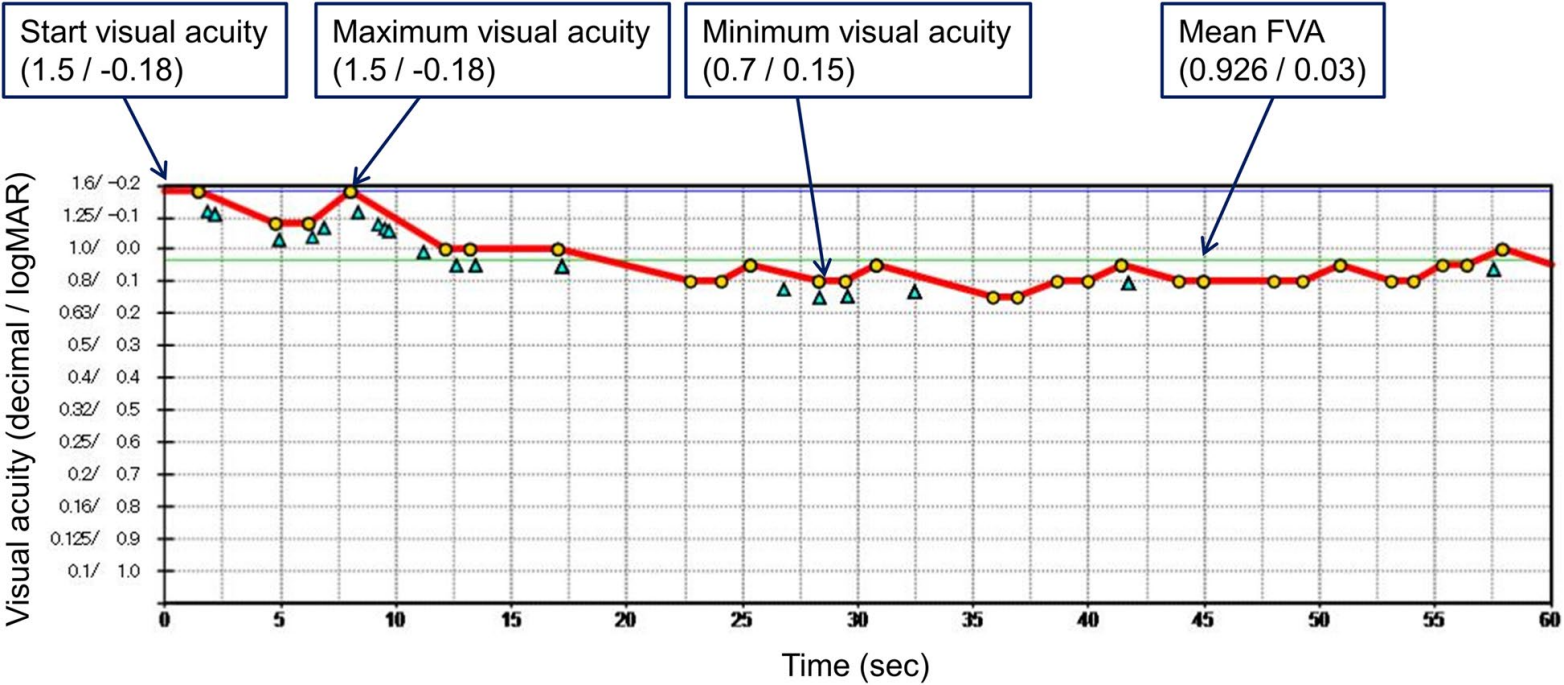
FVA record of a typical eye of a female patient (age: 79) with EDOF IOL. The FVA value is shown with a green line (20/21.6 or 0.03 logMAR). Blinks are indicated with 19 triangles. Visual acuity is presented as decimal/logMAR

Figure 3 is a record of FVA testing with a typical eye of a female patient (age: 79) with EDOF IOL. The initial visual acuity was 20/13 (− 0.18 logMAR), and the visual acuity varied over 60 s. The maximum and minimum visual acuities were 20/13 (− 0.18 logMAR) and 20/30 (0.15 logMAR), and the FVA values during 60 s (green line in Fig. 3) were 20/21.6 (0.03 logMAR). There were 19 blinks which is depicted as triangles.

Table 2 compares the FVA parameters between the EDOF and monofocal IOLs. At 3 months postoperatively, 10 of 17 eyes with the EDOF IOLs and 7 of 27 eyes with the monofocal IOLs were topically treated for DES. Among them, SPK was found in one eye each. There was no eye with cystoid macular edema and other retinal abnormality. No difference was found, except for in the mean response time (*P* = 0.037, t-test). The mean difference in the response time was 0.06 s, while the SDs measured with elder (over 60 years) subjects were 0.12 s [17], so that the difference was consider as clinically negligible. For eyes with and without DES treatments, no difference was observed between the 2 IOLs (*P* > 0.070).

**Table 2:** FVA parameters in eyes with EDOF and monofocal IOLs for all eyes and eyes with and without DES treatments

IOL	EDOF	Monofocal	*P* value
Start visual acuity, logMAR	−0.114 (0.086) [− 0.18 – 0.16]	−0.109 (0.056) [− 0.18 – 0.00]	0.41*
Without DES treatment	−0.109 (0.093) [− 0.18 – 0.15]	−0.100 (0.056) [− 0.18 – 0.00]	0.30*
With DES treatment	−0.121 (0.075) [− 0.18 – 0.00]	−0.135 (0.052) [− 0.18 – − 0.08]	0.82*
FVA value, logMAR	0.011 (0.093) [− 0.13 – 0.17]	0.026 (0.117) [− 0.14 – 0.38]	0.84*
Without DES treatment	− 0.006 (0.096) [− 0.13 – 0.14]	0.022 (0.092) [− 0.14 – 0.21]	0.33*
With DES treatment	0.040 (0.085) [− 0.10 – 0.17]	0.038 (0.179) [− 0.13 – 0.38]	0.43*
VMR	96% (2%) [92 – 100%]	95% (4%) [80 – 101%]	0.68^#^
Without DES treatment	96% (2%) [92 – 100%]	96% (3%) [90 – 101%]	0.49^#^
With DES treatment	94% (2%) [92 – 97%]	94% (7%) [80 – 100%]	0.83^#^
Mean response time, sec	1.40 (0.11) [1.13 – 1.61]	1.46 (0.10) [1.22 – 1.62]	0.037^#^
Without DES treatment	1.39 (0.10) [1.23 – 1.61]	1.46 (0.10) [1.22 – 1.62]	0.070^#^
With DES treatment	1.30 (0.14) [1.13 – 1.58]	1.47 (0.09) [1.32 – 1.60]	0.26^#^
Number of blinks	11.4 (7.8) [1 – 31]	11.5 (9.8) [0 – 41]	0.98^#^
Without DES treatment	9.0 (5.8) [1 – 24]	11.5 (11.0) [0 – 41]	0.39^#^
With DES treatment	15.5 (9.5) [3 – 31]	11.6 (5.4) [6 – 21]	0.30^#^

Mean (standard deviation) [range]

*: Man-Whitney test, #: unpaired t-test, *FVA* functional visual acuity, *EDOF* extended depth-of-focus, *IOL* intraocular lens, *VMR* visual maintenance ratio

## Discussions

In the 3-month observation, there was no difference in the CDVA, photopic contrast sensitivity, and FVA values between eyes with the EDOF and monofocal IOLs. Visual functions of eyes with EDOF and monofocal IOLs were previously evaluated in terms of CDVAs and contrast sensitivities [5, 6]. The current results of the static and dynamic examinations supported no difference in visual functions of the 2 types of IOLs. To our knowledge, the FVA after implantation of presbyopia correction IOLs has not been evaluated. FVA testing has been used for evaluating slight differences in visual functions [8–10, 12, 13], and the current results first revealed the comparability in the distance visual functions.

Deterioration of visual function has been concerned in the use of presbyopia correction IOLs, owing to the addition of multifocal optics [18]. Thus, it was assumed that contrast sensitivity and FVA would be degraded in the use of the EDOF IOLs, compared with the use of monofocal IOLs. However, no difference was found in the mean FVA and VMR. The low add-power of the diffractive optics (1.75 D) would reduce the influence. Furthermore, compensation of chronic aberrations by the echelett optics could effectively improve the optical performance [4].

There were some limitations in this study. First, condition of ocular surface was not examined, while the FVA results were sensitive to dry eyes [8, 9, 16]. In the current study, occurrence of SPK and the use of topical treatment for DES were evaluated, and there were no difference between eyes with 2 IOLs with or without the DES treatments. Also, there was no examination of wavefront aberrations. Increases in higher-order aberrations deteriorate FVA values as well as contrast sensitivity [16, 19]. In the current results, there was no difference in either the contrast sensitivity or FVA value, so the influence of ocular surface and higher-order aberrations would be least influenced. More detailed evaluation would be necessary to confirm the comparability of visual functions after the 2 types of IOLs. Next, only photopic visual function was assessed. Testing of mesopic contrast sensitivity was available with the instrument used, although the current FVA system could measure under photopic conditions only. Under mesopic conditions, visual function declines, and the stability of vision is degraded [20]. It is interesting to evaluate the mesopic visual function of eyes with EDOF IOLs using a newly developed system, since some subjective photic phenomena, such as halos and glare, are seen in mesopic conditions. Lastly, the sample size was limited. The detection power for 27 subjects each was calculated to be 0.986, so that the sample size would be statistically enough for the FVA evaluation. A difference in VMR values was detected with the sample size of 29 patients, when the influence of the increase of surface scattering on particular IOLs was evaluated [13]. Further examinations with larger sample size is desirable for more precise evaluations.

## Conclusion

In summary, the dynamic evaluation of FVA of eyes with diffractive EDOF IOLs indicated that there was no difference in the photopic and distance visual functions of eyes with monofocal IOLs.

## Supplementary Information

**Additional file 1.**

## Data Availability

The dataset is included within the additional file.

## Abbreviations

EDOF: Extended depth-of-focus
IOL: Intraocular lens
FVA: Functional visual acuity
CDVA: Corrected distance visual acuity
MRSE: Manifest refraction spherical equivalent
logMAR: Logarithm of the minimum angle of resolution
AULCSF: Area under the logarithmic contrast sensitivity function
VMR: Visual maintenance ratio
